# Acute ischemic stroke in young adults with tuberculous meningitis

**DOI:** 10.1186/s12879-019-4004-5

**Published:** 2019-04-30

**Authors:** Liming Zhang, Xiaoyu Zhang, Huaqiang Li, Gang Chen, Meijia Zhu

**Affiliations:** 1 Department of Pulmonary and Critical Care Medicine, Chongqing General Hospital, University of Chinese Academy of Science, Chongqing, 404100 China; 2 Department of Neurology, Qianfoshan Hospital, Shandong University, Jinan, 250014 China; 3 Imaging department, Chongqing General Hospital, University of Chinese Academy of Science, Chongqing, 404100 China

**Keywords:** Tuberculous meningitis, Stroke, Risk factor

## Abstract

**Background:**

Ischemic stroke is a common complication in patients with tuberculous meningitis (TBM), which is associated with poor clinical outcome. However, risk factors of stroke in TBM patients were not fully understood, especially in those young adults. Therefore, the aim of our study was to identify risk factors for acute ischemic stroke in young adults with TBM.

**Methods:**

TBM patients (18 to 50 years) without cerebral vascular risk factors were prospective recruited between Feb 2014 and Dec 2017. Patients were defined as stroke group and non-stroke group by brain magnetic resonance imaging (MRI). Demographic characteristics, clinical presentations, cerebrospinal fluid (CSF) examination, basal meningeal enhancement, hydrocephalus, tuberculoma and clinical outcome were compared between two groups. Binary logistic regression was performed to determine risk factors for acute ischemic stroke in young TBM patients.

**Results:**

Fifty-two patients with TBM were included and 12 (23.1%) patients were in stroke group. Patients in stroke group were older. Clinical presentations were comparable between two groups except headache was more common in TBM patients with stroke. In CSF examination, TBM patients with stroke had higher CSF white blood cell. By MRI, patients in stroke group were more likely to have basal meningeal enhancement but less likely to present tuberculoma. Compared to non-stroke group, patients in stroke group had worse short-term clinical outcome. In logistic regression, age (OR = 1.297; 95%CI 1.067, 1.576), CSF white blood cell (OR = 1.023; 95%CI 1.005, 1.042) and basal meningeal enhancement (OR = 23.913; 95%CI 1.398, 408.975) were independent risk factors for stroke. However, tuberculoma (OR = 0.005; 95%CI 0.000, 0.254) was negative related with stroke.

**Conclusions:**

About a quarter of young adults with TBM have acute ischemic stroke which may lead to poor clinical outcome. Age, CSF white blood cell and basal meningeal enhancement are risk factors for acute ischemic stroke in young adults with TBM.

## Background

Tuberculous meningitis (TBM) is one of the most devastating presentations of tuberculosis (TB), which constitutes about 10% of all TB cases and is responsible for about 40% of the deaths of TB in developing countries [1]. The main complications of TBM include cerebral stroke, hydrocephalus and tuberculoma formation [2]. It has been reported that incidence of stroke is about 13–57% in TBM patients, which can cause poor clinical outcome [3]. The mortality is about 3 times higher in TBM patients with stroke compared to those without [3]. Therefore, prevention of ischemic stroke is great important to TBM patients. However, risk factors of stroke were not fully understood in TBM patients, especially in those young adults.

Previous study showed that stage of TBM, basal meningeal enhancement, hydrocephalus, exudate and hypertension were related to stroke in TBM patients [4]. However, all of them were not identified as independent risk factor in regression analysis. The negative result may be related to their included subjects as 33.6% patients were older than 40 years and some of them had cerebral vascular risk factors such as diabetes mellitus and hypertension. By contrast to stroke in older patients, young stroke patients often have many different causes and risk factors. According to the Trial of Org in Acute Stroke Treatment (TOAST) criteria, only 10–15% of strokes in young patients are large-artery atherosclerosis (LAA) which is the main cause of stroke in older patients. In pathological studies of TBM patients, vasculitis and intimal proliferation have been considered to contribute to cerebral vessel damage and cause brain infarcts, while the effect of thrombosis is still uncertain [5]. Therefore, the exact mechanisms of stroke may have difference between young and elder TBM patients and it may be reasonable to investigate risk factor of stroke in TBM patients by different age groups. In this study, we only focused on young TBM patients aged 18–50 years. Patients with cerebral vascular risk factors were excluded in our study in order to decrease the impact of atherosclerosis.

The aim of our study was to identify independent risk factors for acute ischemic stroke in young TBM patients. Short-term clinical outcome of young TBM patients were also investigated.

## Methods

### Subject population

This was a prospective study and we recruited TBM patients from Chongqing General Hospital between Feb 2014 and Dec 2017. Chongqing General Hospital has a tuberculosis department for TB patients. Patients fulfilled the following inclusion criteria were included: 1) age 18–50 years; 2) diagnosed as TBM; 3) brain magnetic resonance imaging (MRI) data available; 4) available written informed consent. Patients with cerebral vascular risk factors, including history of hypertension, diabetes mellitus, hyperlipidaemia, coronary heart disease, atrial fibrillation, cerebral ischemic stroke and hemorrhage, were excluded. Patients with history of cerebral artery dissection, moyamoya disease, patent foramen ovale, rheumatic valvular heart disease, post-cardiac surgery or catheter intervention and hematologic hypercoagulable state were also excluded. The study was approved by the ethics committees of Chongqing General Hospital, China.

### Diagnostic criteria for tuberculous meningitis

TBM was diagnosed according to a standard case definition published in 2010 [6]. Patients were defined as ‘definite’ TBM if acid-fast bacilli seen in the cerebrospinal fluid (CSF); or *Mycobacterium tuberculosis* cultured from the CSF; or a CSF positive commercial nucleic acid amplification test. Patients were defined as ‘probable’ TBM if their diagnostic score ≥ 12 (at least 2 points either come from CSF or cerebral imaging criteria) plus exclusion of alternative diagnoses. Patients were defined as ‘possible’ TBM if their diagnostic score were 6 to 11 plus exclusion of alternative diagnoses.

### Demographic and clinical assessment

The clinical information collected included age, sex, history of smoking and infection of human immunodeficiency virus (HIV). Clinical presentations including fever, headache, vomiting, altered consciousness, visual disturbance, cranial nerve palsy, focal weakness, neck stiffness and seizure were recorded at admission. Glasgow Coma Scale (GCS) was used to assess the severity of consciousness. Severity of TBM was graded into: stage I, (GCS: 15) alert and oriented without focal neurological deficit; stage II, (GCS: 10–14) with or without focal neurological deficit or (GCS: 15) with focal neurological deficit; and stage III, GCS less than 10 with or without focal neurological deficit [1]. CSF was examined for cells, protein, glucose and chloride, and which was also subjected to pathogenic culture.

### Cerebral imaging and diagnosis of stroke

Cerebral MR examination were conducted in all patients at admission, and repeated if patient had new neurological symptoms and signs during treatment. Brain MRIs were acquired on the same 1.5 T scanner. The parameters of MR examination were as follows: axial T2-weighted (repetition time 4500 ms; echo time 93 ms), axial T1-weighted imaging (repetition time 2000 ms; echo time 9.2 ms), axial diffusion weighted imaging (DWI) (repetition time 3300 ms; echo time 91 ms), and axial fluid-attenuated inversion recovery (FLAIR) sequences (repetition time 8000 ms; echo time 86 ms). All the above-mentioned sequences had 5 mm slice thickness and 1.5 mm interslice gap. T1 contrast images were also obtained.

**Fig. 1:**
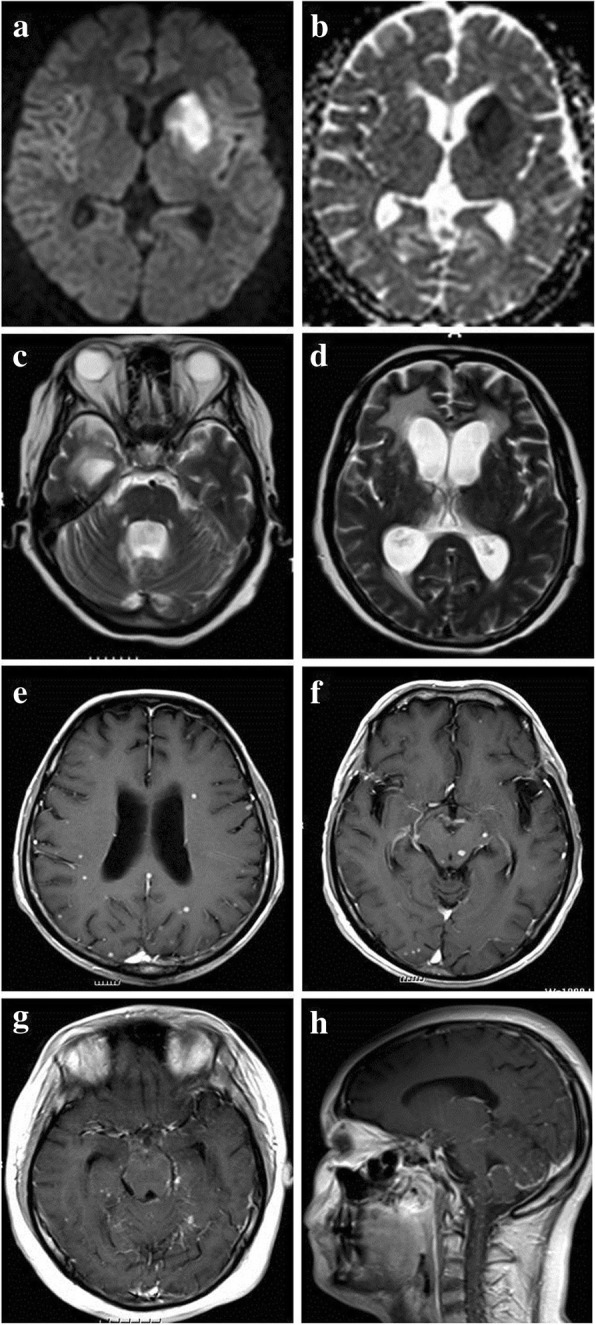
MR presentations of patients with tuberculous meningitis. **a**-**b**TBM patient with acute ischemic stroke in basal ganglia (hyperintense in DWI and hypointense in ADC); **c**-**d** TBM patient with hydrocephalus; **e**-**f**:TBM patient with multiple tuberculomas; **g**-**h** TBM patient with basal and cerebellar meningeal enhancement

The presence of acute ischemic stroke, basal meningeal enhancement, hydrocephalus and tuberculoma were identified by an experienced neuroradiologist and a neurologist who were blinded to their clinical information (Fig. 1). Discrepancy in readings was resolved by consensus. The diagnosis of acute ischemic stroke was based on hyperintense on DWI and hypointense on apparent diffusion coefficient (ADC). Patients were defined as stroke group and non-stroke group by their MRI findings.

### Clinical outcome assessment

All patients received routine anti-tuberculosis treatment according to recommendations of the World Health Organization (Fourth edition). Disability status was assessed by Modified Rankin Scale (mRS) at discharge. Short-term clinical outcome was defined as good outcome (mRS: 0); intermediate outcome (mRS: 1–2); and poor outcome (mRS: 3–5 or death) [7].

### Statistic analysis

All statistics were presented as mean and SD for continuous variables with normal distribution, median and interquartile range for continuous variables with non-normal distribution, frequency and percentages for categorical variables. Univariate analysis was performed between stroke group and non-stroke group. Continuous variables with normal distribution were compared with Student t test with significance set at *p* < 0.05, while Wilcoxon rank sum test for continuous variables with non-normal distribution. Categorical variables were compared by means of x^2^ test or fisher’s exact, Wilcoxon rank sum test. Binary logistic regression was performed to determine risk factors of stroke in TBM patients.

## Results

During study period, 80 patients were diagnosed as TBM. Twenty-eight patients were excluded because of overage (16 patients), unavailable MRI data (5 patients), history of stroke (3 patients) and hypertension (4 patients). Finally, 52 patients were included and 12 (23.1%) patients had acute ischemic stroke. The average age was 30.3 ± 9.9 years and 33 (63.5%) patients were male. 6 (11.5%) patients were defined as definite TBM, 40 (76.9%) patients were probable TBM and 6 (11.5%) patients were possible TBM. 3 (5.8%) patients were HIV infected. At admission, 36 (69.2%) TBM patients were in stage I and only 1 (1.9%) patients had GCS < 8. In stroke group, 7 (58.3%) patients were symptomatic stroke and 5 (41.7%) patients were silent stroke. 9 (75%) patients had single stroke and 6 (50%) patients had stroke located in basal ganglia. The majority of patients had good clinical outcome after treatment, while 8 (15.4%) patients had poor clinical outcome and 3 (5.8%) patients were dead.

Compared to patients in non-stroke group, patients in stroke group were older (35.9 ± 10.8 years vs 28.4 ± 9.3 years, *p* = 0.030) (Table 1). There was no significant difference in clinical presentations between two groups except that patients with stroke were more likely to have headache (100% vs 67.5%, *p* = 0.024). In CSF examination, patients with stroke had higher level of white blood cell (median 83/μl vs 20/μl, *p* = 0.022). No differences were found in CSF protein, glucose and chloride (Table 2). By MRI, patients in stroke group were less likely to present tuberculoma (25.0% vs 70.0%, *p* = 0.014). Basal meningeal enhancement was more common in patients with stroke compared to those without stroke, but without significant difference (66.7% vs 35.0%, *p* = 0.051). At discharge, patients with ischemic stroke had worse clinical outcome (poor outcome: 33.3% vs 10%, *p* = 0.024).

**Table 1:** Characteristics between young TBM patients in stroke group and non-stroke group

	Total (*n* = 52)	Stroke group (*n* = 12)	Non-stroke group (*n* = 40)	*p* value
Age (years)	30.3 ± 9.9	35.9 ± 10.8	28.4 ± 9.3	0.040
Sex (male, %)	33 (63.5%)	8 (66.7%)	25 (62.5%)	1.000
Smoking, %	11 (21.2%)	2 (25.0%)	9 (22.5%)	1.000
HIV, %	3 (5.8%)	2 (16.7%)	1 (2.5%)	0.254
Clinical presentations				
Fever, %	44 (84.6%)	10 (83.3%)	34 (85.0%)	1.000
Headache, %	39 (75.0%)	12 (100%)	27 (67.5%)	0.024
Vomiting, %	17 (32.7%)	5 (41.7%)	12 (30.0%)	0.686
Altered consciousness, %	17 (32.7%)	5 (41.7%)	12 (30.0%)	0.686
Visual disturbance, %	6 (11.5%)	2 (16.7%)	4 (10.0%)	0.905
Cranial nerve palsy, %	8 (15.4%)	4 (33.3%)	4 (10.0%)	0.131
Focal weakness, %	12 (23.1%)	5 (41.7%)	7 (17.5%)	0.176
Neck stiffness, %	21 (40.4%)	7 (58.3%)	14 (35.0%)	0.267
Seizure, %	8 (15.4%)	2 (16.7%)	6 (15.5%)	1.000
GCS score				
3–10	7 (13.5%)	4 (33.3%)	3 (7.5%)	
11–15	45 (86.5%)	8 (66.7%)	37 (92.5%)	0.069
Stage of TBM				
Stage I	36 (69.2%)	7 (58.3%)	29 (72.5%)	
Stage II	9 (17.3%)	1 (8.3%)	8 (20.0%)	
Stage III	7 (13.5%)	4 (33.4%)	3 (7.5%)	0.195
Clinical outcome				
Good outcome (mRS = 0)	35 (67.3%)	5 (41.7%)	30 (75.0%)	
Intermediate outcome (mRS 1–2)	9 (17.3%)	3 (25.0%)	6 (12.5%)	
Poor outcome (mRS 3–5 and death)	8 (15.4%)	4 (33.3%)	4 (10.0%)	0.024

TBM indicates tuberculous meningitis; HIV indicates human immunodeficiency virus; GCS indicates Glasgow Coma Scale; mRS indicates Modified Rankin Scale

**Table 2:** CSF examination and neuroimaging between young TBM patients in stroke group and non-stroke group

	Total (*n* = 52)	Stroke group (*n* = 12)	Non-stroke group (*n* = 40)	*p* value
CSF examination				
white blood cell, /μl ^a^	26 (8, 189)	83 (31, 227)	20 (5, 76)	0.022
Protein, mg/dL ^a^	111 (66, 197)	126 (80, 286)	106 (57, 158)	0.268
Glucose, mmol/L	2.4 ± 1.0	2.2 ± 1.2	2.4 ± 1.0	0.559
Chloride, mmol/L	113.9 ± 6.7	111.5 ± 5.2	114.7 ± 7.0	0.133
Neuroimaging findings				
Basal meningeal enhancement, %	22 (42.3%)	8 (66.7%)	14 (35.0%)	0.051
Hydrocephalus, %	17 (32.7%)	3 (25.0%)	14 (35.0%)	0.767
Tuberculoma, %	31 (59.6%)	3 (25.0%)	28 (70.0%)	0.014

^a^Continuous variables with non-normal distribution are expressed as median (interquartile range). TBM indicates tuberculous meningitis; CSF indicates cerebrospinal fluid

Binary logistic regression was performed to determine risk factors of acute ischemic stroke, with age, TBM stage, CSF white blood cell, basal meningeal enhancement, hydrocephalus and tuberculoma in the model. We found that age (OR = 1.297; 95%CI 1.067, 1.576), CSF white blood cell (OR = 1.023; 95%CI 1.005, 1.042) and basal meningeal enhancement (OR = 23.913; 95%CI 1.398, 408.975) were independent risk factors for acute ischemic stroke. However, tuberculoma (OR = 0.005; 95%CI 0.000, 0.254) were negative related with acute ischemic stroke (Table 3).

**Table 3:** Binary logistic regression for stroke in young TBM patients

Variables	n	Acute ischemic Stroke		
		*β*	*p* value	Exp(B) 95% CI
Age	52	0.260	0.009	1.297 (1.067, 1.576)
CSF white blood cell	52	0.023	0.013	1.023 (1.005, 1.042)
Basal meningeal enhancement	52	3.174	0.028	23.913 (1.398, 408.975)
Tuberculoma	52	−5.211	0.008	0.005 (0.000, 0.254)

TBM indicates tuberculous meningitis; CSF indicates cerebrospinal fluid

## Discussion

Stroke is a common complication of TBM patients, which can cause irreversible brain damage [2]. Results from many studies have demonstrated that stroke predict poor clinical outcome, which may be disastrous consequence for young TBM patients [1, 8–10]. However, risk factors of stroke in young TBM patients were not fully understood. In our study, we found that age, CSF white blood cell and basal meningeal enhancement were independent risk factors for stroke in young TBM patients.

About 13–57% of TBM patients have cerebral ischemic stroke, which is about 22–72% in autopsy [3, 11]. The diversity may depend on evaluation methods. Stroke in TBM patients may develop insidiously and present as asymptomatic or silent stroke. The most common vulnerable brain region was basal ganglia where has been described as ‘tubercular zone’. It is comprised of head of the caudate nucleus, anteriomedial thalami and anterior limb and genu of internal capsule. In a study involving 122 TBM patients, 55 patients had stroke and 54% strokes were located in basal ganglia [4]. Another study had similar results with 49% of infarctions in basal ganglia [12]. In our study, 50% of patients had stroke located in basal ganglia, which is consistent with previous studies. Strokes in ‘tubercular zone’ may be associated with basilar exudates. Dense fibrinocellular exudates may wrap middle cerebral trunks and their penetrating branches, which may contribute to vasculitis and cause strokes in ‘tubercular zone’.

In clinical presentations, fever and headache are the most common symptoms in TBM patients, which were also confirmed in our young TBM patients [8, 10, 13]. Altered consciousness has been reported about 17–69% in TBM patients, which was 32.7% in our study [8, 10, 13]. TBM patients with stroke were more likely to have focal weakness [8, 10]. We found that 41.7% patients in stroke group had focal weakness, which was 17.5% in non-stroke group. This comparison did not reach significant difference, which may be related to our small sample size. Cranial nerve palsy is also an important symptom in TBM patients which is mainly caused by gelatinous exudates in the basilar regions of the brain or increased intracranial pressure [14].

Studies of stroke in TBM patients were limited and their predictors were still uncertain. In a previous study, hypertension, stage of meningitis, presence of hydrocephalus and exudates were related to stroke in TBM patients [4]. However, they disappeared in regression analysis. It has been accepted that stroke in TBM patients was mainly caused by vasculitis secondary to the meningeal inflammation, which can be classified into three patterns: 1) infiltrative, 2) proliferative and 3) necrotizing vascular lesions [3]. Duration of TBM may determine the relative frequency of infiltrative, proliferative and necrotizing changes in the cerebral vessels. The contribution of atherosclerosis to stroke in TBM patients is still controversial as evidence of cerebral arterial thrombosis is uncommon in autopsy studies. However, atherosclerosis aggravates with age and thrombosis is the main cause of stroke in elder population. Therefore, it was difficult to determine whether stroke was caused by vasculitis or atherosclerosis in elder TBM patients, especially in those with cerebral vascular risk factors. In our study, elder patients and those with cerebral vascular risk factors were excluded, reducing the effects of atherosclerosis. Our results showed that CSF white blood cell and basal meningeal enhancement were independent risk factors for stroke in young TBM patients, supporting the contribution of inflammatory reaction.

Tuberculoma has been reported in 16–40% of TBM patient, which may be a benign condition with good prognosis [1]. The majority of the cerebral tuberculomas were supratentorial located and multiple tuberculomas were much more frequent than single lesion [7]. Relationship between tuberculoma and cerebral infarction is still controversy. Results from two studies showed that presence of tuberculoma were similar between patients with stroke and those without [4, 7]. In another study involving 65 children with TBM, the incidence of tuberculoma was 40% in patients without infarct, which was only 8% in those with infarct [15]. Research involving 404 TBM patients also showed similar results [1]. In our study, we found that tuberculoma was more common in non-stoke group, which is consistent with previous studies. Tuberculoma most likely represents an immune response to tuberculous brain parenchymal infections, which may restrict tissue mycobacterial growth. However, the underlying mechanisms between tuberculoma and stroke are still unknown and more researches are needed.

TBM patients with stroke have poor clinical outcome. All TBM patients should be managed with standard anti-tubercular therapy and supportive treatments. Many studies have shown anti-platelet therapy is beneficial for TBM patients with stroke because of prothrombotic state. In other studies, TBM patients treated with corticosteroids may have favorably outcome because of anti-inflammatory effect.

The strength of our study included the homogeneity of our recruited young patients. Several limitations should be noted in our study. First, our study was conducted in single center with inevitable selected bias. Second, brain magnetic resonance angiography (MRA) was not analyzed in our study as insufficient data, which may be related with stroke. It has been reported that about 40% of TBM patients have abnormal MRA [16]. Third, our sample size was small and only young patients were included which reduce the external validity of our study.

## Conclusions

About a quarter of young adults with TBM have acute ischemic stroke which may be related with poor clinical outcome. Age, CSF white blood cell and basal meningeal enhancement can predict the occurrence of acute ischemic stroke in young adults with TBM.

## Abbreviations

ADC: Apparent diffusion coefficient
CSF: Cerebrospinal fluid
DWI: Diffusion weighted imaging
FLAIR: Fluid-attenuated inversion recovery
GCS: Glasgow Coma Scale
HIV: Human immunodeficiency virus
MRI: Magnetic resonance imaging
mRS: Modified Rankin Scale
TB: Tuberculosis
TBM: Tuberculous meningitis
